# Supplementary material for: Floating nurseries? Scyphozoan jellyfish, their food and their rich symbiotic fauna in a tropical estuary
Source: PeerJ. 2018 Jun 19;6:e5057. doi: 10.7717/peerj.5057 (PMC6014317; doi:10.7717/peerj.5057)
Supplement: Table S1 — Significant factors ( α = 0.05) are highlighted in bold. [file peerj-06-5057-s002.docx]

Supplemental Table S1. Results of A) the PERMANOVA analysis on the differences in the structure of the diet of *Stomolophus meleagris* among months and seasons and B) pair-wise tests for differences between pairs of months in each season. Significant factors (α = 0.05) are highlighted in bold.

| A | Source | df | SS | MS | Pseudo-*F* | P(perm) | Unique perms |
| --- | --- | --- | --- | --- | --- | --- | --- |
|  | Season | 1 | 12562 | 12562 | 2.6554 | 0.128 | 890 |
|  | Month(season) | 5 | 24708 | 4941 | 2.4473 | **0.002** | 998 |
|  | Res | 105 | 212020 | 2019 |  |  |  |
|  | Total | 111 | 250000 |  |  |  |  |
| B | Months |  | Season |  | *T* | P(perm) | perms |
|  | February, March | | 1 |  | 1.010 | 0.407 | 999 |
|  | February, April | | 1 |  | 1.514 | 0.059 | 998 |
|  | February, May | | 1 |  | 1.564 | **0.043** | 998 |
|  | March, April |  | 1 |  | 1.620 | **0.039** | 999 |
|  | March, May |  | 1 |  | 1.415 | 0.100 | 998 |
|  | April, May |  | 1 |  | 1.002 | 0.415 | 999 |
|  | February, March | | 2 |  | 2.144 | **0.006** | 998 |
|  | February, April | | 2 |  | 1.908 | **0.012** | 997 |
|  | March, April |  | 2 |  | 1.289 | 0.150 | 998 |
